# Prognostic Factors on the Graft-versus-Host Disease-Free and Relapse-Free Survival after Adult Allogeneic Hematopoietic Stem Cell Transplantation

**DOI:** 10.1155/2016/5143071

**Published:** 2016-03-30

**Authors:** Yao-Chung Liu, Sheng-Hsuan Chien, Nai-Wen Fan, Ming-Hung Hu, Jyh-Pyng Gau, Chia-Jen Liu, Yuan-Bin Yu, Liang-Tsai Hsiao, Tzeon-Jye Chiou, Cheng-Hwai Tzeng, Po-Min Chen, Jin-Hwang Liu

**Affiliations:** ^1^Division of Hematology, Department of Medicine, Taipei Veterans General Hospital, Taipei 11217, Taiwan; ^2^Faculty of Medicine, National Yang-Ming University, Taipei 11221, Taiwan; ^3^Division of Hematology and Oncology, Department of Medicine, Taipei Veterans General Hospital, Taitung Branch, Taitung City, Taitung County 95059, Taiwan; ^4^Institute of Clinical Medicine, National Yang-Ming University, Taipei 11221, Taiwan; ^5^Department of Ophthalmology, Taipei Veterans General Hospital, Taipei 11217, Taiwan; ^6^Department of Medicine, Cardinal Tien Hospital, New Taipei City 23148, Taiwan

## Abstract

The cure of hematologic disorders by allogeneic hematopoietic stem cell transplantation (HSCT) is often associated with major complications resulting in poor outcome, including graft-versus-host disease (GVHD), relapse, and death. A novel composite endpoint of GVHD-free/relapse-free survival (GRFS) in which events include grades 3-4 acute GVHD, chronic GVHD requiring systemic therapy, relapse, or death is censored to completely characterize the survival without mortality or ongoing morbidity. In this regard, studies attempting to identify the prognostic factors of GRFS are quite scarce. Thus, we reviewed 377 adult patients undergoing allogeneic HSCT between 2003 and 2013. The 1- and 2-year GRFS were 40.8% and 36.5%, respectively, significantly worse than overall survival and disease-free survival (log-rank *p* < 0.001). European Group for Blood and Marrow Transplantation (EBMT) risk score > 2 (*p* < 0.001) and hematologic malignancy (*p* = 0.033) were poor prognostic factors for 1-year GRFS. For 2-year GRFS, EBMT risk score > 2 (*p* < 0.001), being male (*p* = 0.028), and hematologic malignancy (*p* = 0.010) were significant for poor outcome. The events between 1-year GRFS and 2-year GRFS predominantly increased in relapsed patients. With prognostic factors of GRFS, we could evaluate the probability of real recovery following HSCT without ongoing morbidity.

## 1. Introduction

The treatment outcome of allogeneic hematopoietic stem cell transplantation (HSCT) for hematological disorders was determined by two major factors: transplant-related morbidity or mortality (TRM) and disease relapse [1]. There are many issues of dilemma in the management of patients receiving allogeneic HSCT. Graft-versus-host disease (GVHD) is the major cause of TRM while GVHD-associated graft-versus-leukemia (GVL) effect can reduce the risk of malignancy relapse [2]. It has been well demonstrated that T-cell depletion of the allograft can reduce GVHD risk and therefore lower TRM [3]; however, this benefit is traded off by the risk of graft rejection and disease relapse. Regarding the intensity of conditioning, myeloablative conditioning regimen has lower risk of disease relapse while it increases risk of nonrelapse mortality (NRM), especially during first 100 days, as compared with reduced-intensity conditioning (RIC) [4, 5]. Conversely, although RIC with fludarabine-based regimen has been used as a substitute in allowing older patients and those with comorbidities to safely undergo transplantation, relapse rates and GVHD risk were higher [6, 7]. It is often a difficult task to keep balance between control of posttransplant GVHD and the risk of disease relapse. Life-threatening posttransplant complications related to GVHD would increase NRM [8, 9]. Efforts at reducing GVHD with higher doses of immunosuppressive agents and steroids can also lead to excess deaths from opportunity infections, such as invasive fungal or viral infection [10–12]. It is mandatory to evaluate various pretransplant, peritransplant, and posttransplant risk factors that would influence survival in each individual being considered to undergo the procedure. The European Group for Blood and Marrow Transplantation (EBMT) risk score offers a simple tool to approach immediately pretransplant risks of HSCT [13]. This scoring system has been demonstrated to correlate with NRM, relapse risk, and overall survival after allogeneic HSCT for hematological disorders [13]. However, the current evaluation of clinical outcome focusing on survival alone cannot fully reflect the long-lasting complications associated with transplantation, especially GVHD, and no single evaluation can completely characterize cure without ongoing morbidity.

To address this issue, composite endpoints for posttransplant survival proposed by the Blood and Marrow Transplant Clinical Trials Network are applied for HSCT patients [14–16]. The novel composite endpoints include grades 3-4 acute GVHD (aGVHD) and chronic GVHD (cGVHD) requiring systemic treatment, relapse, or death, and the outcome was defined as GVHD-free/relapse-free survival (GRFS) [14, 15]. To the best of our knowledge, only one article described the clinical prognostic factors and characteristics of GRFS at one year among different age groups (≥21 or <21 years) [16]. To further understand the real recovery from HSCT without major complication, we retrospectively reviewed 377 adult patients with hematological disorders treated with allogeneic HSCT between 2003 and 2013 in our institute and evaluated overall GRFS, overall survival (OS), and disease-free survival (DFS) at 1 and 2 years. In addition, we also used EBMT risk score to predict GRFS and tried to identify other prognostic factors influencing survival at the first 12 and 24 months. With the information, we hoped to optimize treatment outcome of the patients undergoing allogeneic HSCT.

## 2. Materials and Methods

### 2.1. Study Patient Population

Adult patients (age ≥ 18 years) receiving allogeneic HSCT between January 2003 and September 2013 in our institute were recruited for analysis. All patients had been regularly followed till September 2014. Pertinent clinical data including age, gender, disease diagnosis, comorbidities, type of transplant, human leukocyte antigen (HLA) matching, conditioning regimen, GVHD, and other clinical complications were collected. DFS was defined as the time from last transplantation to relapse of the underlying disease or death; OS was defined as the time from last transplantation to death. In addition, the clinical outcome was also evaluated using a new composite endpoint of GRFS defined as absence of grade 3-4 aGVHD, systemic immunosuppressive therapy requiring cGVHD, relapse, or death for any causes during one and two years after allogeneic HSCT. The prognostic factors for 1- and 2-year GRFS were also explored. In the components of the GRFS events, all data were recognized as the first posttransplant event within 1 and 2 years. For those undergoing multiple allogeneic transplants, parameters pertinent to GRFS after last transplant were collected and analyzed. The donor source was predominantly peripheral blood stem cell in our study (*n* = 372). The retrospective review of medical records was approved by the institutional ethical committee in agreement with the Helsinki Declaration of 1975, revised in 2008.

### 2.2. Transplant Details and GVHD Prophylaxis

HLA-typing tests of intermediate resolution for 6 or 8 alleles (HLA-A, HLA-B, HLA-DR, or HLA-C) were used to select donors for allogeneic HSCT. Donor's types included matched sibling donor, matched unrelated marrow, related haploidentical donor, or umbilical cord blood. Accordingly, patients were categorized into fully matched group or mismatched group with mismatch in one or more alleles or antigens. Myeloablative conditioning regimens include busulfan (4 mg/kg/day for 4 days) combined with cyclophosphamide (60 mg/kg/day for 2 days) or total body irradiation (TBI) of 1200 cGy combined with cyclophosphamide (60 mg/kg/day for 2 days). Fludarabine-based RIC regimens were administered to patients of old age or with comorbidities.

Standard protocol with cyclosporine (IV 3.0 mg/kg/day in 2 split doses with adjusted trough plasma level maintained at 100–250 *μ*g/L) and short-term low dose methotrexate (15 mg/m^2^ on day +1 and then 10 mg/m^2^ on days +3, +6, and +11 after HSCT) were adopted for GVHD prophylaxis. In addition, recipients of unrelated donor transplants also received rabbit anti-thymocyte globulin (2 mg/kg/day for 2-3 days). Prophylactic antiviral therapy during transplantation was not routine to administration, because the seropositive rate of cytomegalovirus (CMV) IgG in Taiwan was very high [17] and most cases were “seropositive donors transfusion to seropositive donors.” Instead, a policy of preemptive therapy was adopted. We detected the CMV viremia by real time PCR after transplantation and ganciclovir therapy was initiated when CMV copy numbers increased significantly or CMV disease developed clinically. In addition, trimethoprim-sulfamethoxazole was prescribed within 3 months after engraftment and in parallel with immunosuppressive therapy for GVHD. Severity of aGVHD was graded according to the system of Glucksberg and Thomas and severity of the cGVHD was determined by NIH scoring system [18, 19]. Patients with aGVHD > overall grade 2, extensive cGVHD, or GVHD-related lung disease would receive treatment with methylprednisolone (MTP) 1-2 mg/kg/day.

### 2.3. Transplantation Risk Evaluation

We calculated transplantation risk based on EBMT risk scoring system [13] according to the age at HSCT, disease stage prior to transplantation, time interval from diagnosis to transplant, donor types, and donor recipient sex combination. The EBMT risk score was also used as a variable factor in our analysis.

### 2.4. Statistical Analysis

Kaplan-Meier product-limit method was used for evaluation of 1- and 2-year posttransplant OS, DFS, and GRFS. The log-rank test was used to compare survival curves. We also explored potential prognostic factors of GRFS including age, gender, underlying disease diagnosis, EBMT risk score, transplant type, conditioning therapy, pretransplant comorbidities and 1- or 2-year posttransplant CMV reactivation, and posttransplant lymphoproliferative disease (PTLD). The posttransplant CMV reactivation indicated that antiviral agents administration was necessary based on the increased CMV copy numbers detected by real time polymerase chain reaction or clinical symptoms. The prognostic factors were analyzed using Cox proportional hazard models. Factors with statistical significance (*p* < 0.05) upon univariate analysis were included in multivariate analysis. All statistically significant levels were set at *p* < 0.05. Results were expressed as hazard ratio (HR) and their corresponding 95-percent confidence intervals (95% CI). In the analysis of age as prognostic factor, age of 35 years was adopted as cutoff value based on the results of receiver operating characteristic curve. All calculations were performed using the Statistical Package of Social Sciences software (version 18.0; SPSS Inc., Chicago, IL, USA).

## 3. Results

We retrospectively reviewed totally 377 patients receiving allogeneic HSCT between January 2003 and September 2013. The median age at HSCT was 41 years (range: 18–67). Male patients were slightly predominant (55.4%). Hematological malignancies comprised 87.3% (*n* = 329) of diagnosis and the rest were other nonmalignant diseases (12.7%, *n* = 48, including 45 with severe aplastic anemia and 3 with myelodysplastic syndrome without excess blasts). Donor types included 181 matched sibling donors (48.0%) and 196 patients (52.0%) with matched unrelated donors or incompletely matched sibling and unrelated sources. Median duration of follow-up after HSCT was 554 days (range: 11–4097). Myeloablative conditioning regimen was more frequently used in the cohort (65.8%). About half of the patients experienced posttransplant CMV reactivation within one year (49.9%). Eventually, 10 patients had CMV pneumonitis, 2 had CMV enteritis, and 2 had CMV retinitis. Few patients suffered from PTLD (3.7% and 4.0% within one and two years). The clinical characteristics were detailed in Table 1.

### 3.1. Clinical Prognostic Factors for GRFS

In the univariate regression analysis for prognostic factors, age at HSCT (*p* = 0.011; HR: 1.446), EBMT risk score (*p* < 0.001; HR: 2.197), disease type at diagnosis (*p* = 0.002; HR: 2.244), gender (*p* = 0.012; HR: 1.419), use of fludarabine-based conditioning regimen (*p* = 0.012; HR: 1.491), and posttransplant CMV reactivation (*p* = 0.033; HR: 1.335) significantly impacted 1-year GRFS (Table 2). In multiple regression analysis, the prognostic factors that remained significant for GRFS at 1 year were EBMT risk score > 2 (*p* < 0.001; HR: 1.897; 95% CI: 1.385–2.599) and hematologic malignancy at diagnosis (*p* = 0.033; HR: 1.763; 95% CI: 1.048–2.966).

For 2-year prognostic factors of GRFS, age at HSCT (*p* = 0.008; HR: 1.452), EBMT risk score (*p* < 0.001; HR: 2.165), disease type at diagnosis (*p* < 0.001; HR: 2.495), gender (*p* = 0.005; HR: 1.456), fludarabine-based conditioning regimen (*p* = 0.009; HR: 1.494), and posttransplant CMV reactivation (*p* = 0.023; HR: 1.349) still significantly impacted 2-year GRFS (Table 3). However, in multiple regression analysis, the poor prognostic factors for 2-year GRFS were EBMT risk score > 2 (*p* < 0.001; HR: 1.835; 95% CI: 1.354–2.486), being male (*p* = 0.028; HR: 1.348; 95% CI: 1.032–1.761), and hematologic malignancy at diagnosis (*p* = 0.010; HR: 1.979; 95% CI: 1.178–3.324).

**Figure 1:**
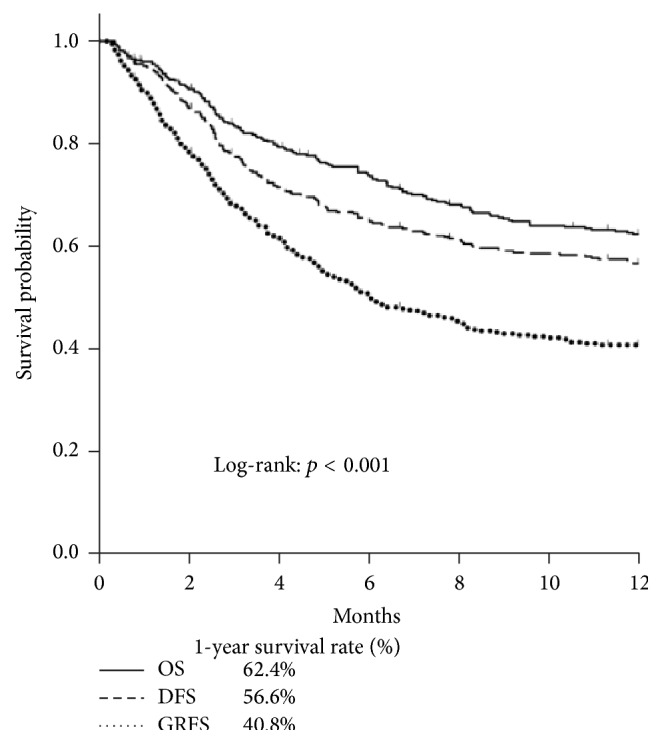
Kaplan-Meier estimates of 1-year GRFS.

**Figure 2:**
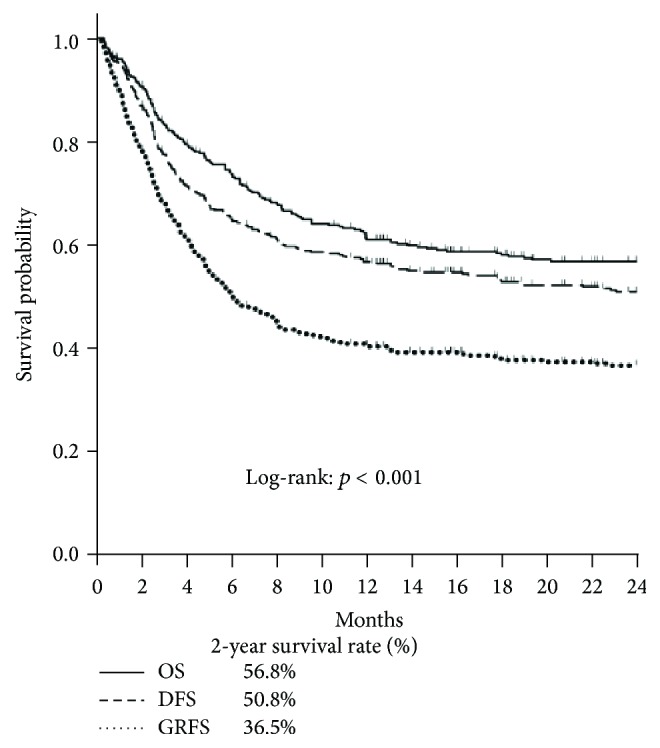
Kaplan-Meier estimates of 2-year GRFS.

**Figure 3:**
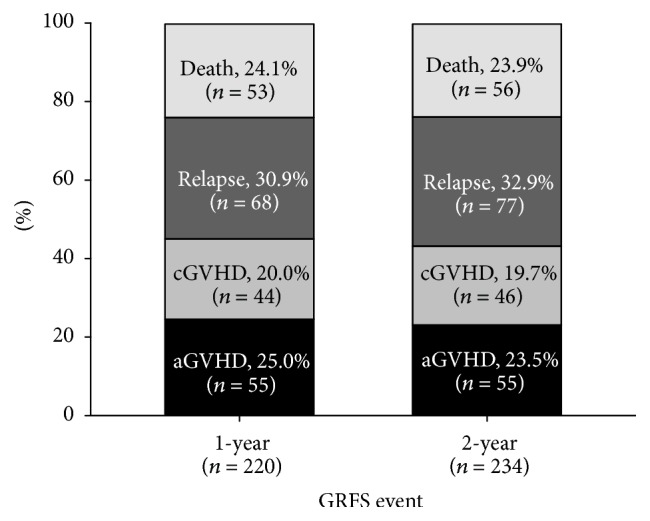
Distribution of individual components between 1-year GRFS and 2-year GRFS.

In the study, the survival rate of Kaplan-Meier curve for 1- and 2-year GRFS revealed 40.8% and 36.5%, respectively, as significantly compared with DFS (1 year: 56.6%; 2 years: 50.8%) and OS (1 year: 62.4%; 2 years: 56.8%) (log-rank *p* < 0.001; Figures 1 and 2). In the four events of 1- and 2-year GRFS, relapsed group was increased prominently in the 2-year GRFS (30.9% to 32.9%; Figure 3). For the poor prognostic factors of GRFS including being male, EBMT risk score > 2, and hematologic malignancy at diagnosis, predominantly increased proportions in relapsed and death subgroups were noted within 1 and 2 years. No marked difference was found between aGVHD and cGVHD within 1 and 2 years (Figure 3).

## 4. Discussion

By using the concept of GRFS defined by Blood and Marrow Transplant Clinical Trials Network, we found that 40.8% and 36.5% of our patients could survive to 1 and 2 years, respectively, without experiencing relapse or significant GVHD. Compared to data from the Center for International Blood and Marrow Transplant Research between 2006 and 2009, our 1-year probability of GRFS was better (40.8% versus 23%) [15]. In comparison to a recent article reported by Holtan et al., the 1-year OS (63%) and DFS (53%) were similar to our cohort (Figure 1); however, our patients had better 1-year GRFS (40.8% versus 31%) and 2-year GRFS of our patients was even similar to 1-year GRFS reported by Holtan et al. (36.5% versus 31%) (Figure 2) [16]. This discrepancy may be ascribed to less chronic GVHD in our patients. Generally speaking, only 30 to 40% patients receiving allogeneic HSCT could survive to 2 years without major complications or death. Since chronic GVHD could cause significant morbidity, it is obvious that OS and DFS could not fully represent optimal recovery after allogeneic HSCT. The use of a time-to-event analysis for the survival only evaluates the time to first event without reflecting specified composite endpoints of GRFS events.

To the best of our knowledge, only one article described prognostic factors for GRFS in adult patients undergoing allogeneic HSCT [16]. In that study, high-risk disease, initial diagnosis, and year of HSCT between 2000 and 2007 were associated with poor GRFS at 1 year. In our study, age > 35 years at HSCT, EBMT risk score > 2, hematologic malignancy at diagnosis, being male, fludarabine-based conditioning regimen, and posttransplant CMV reactivation revealed significant association with inferior 1-year and 2-year GRFS in univariate regression analysis. However, in multiple regression analysis, EBMT risk score > 2, hematologic malignancy for HSCT, and multiple transplants significantly demonstrated worse 1-year and 2-year GRFS. Additionally, male patients were significantly associated with inferior 2-year GRFS and a trend of inferior GRFS at 1 year. Our study showed results consistent with previous reports by Holtan et al. [16] regarding the prognostic factors of recipient age, advanced disease, and disease risk. However, we found that EBMT risk score provided a reliable practical tool not only to assess the pretransplant risks but also to predict the outcome of GRFS.

Pretransplant risk classification is important for predicting the outcome of patients undergoing allogeneic HSCT. The EBMT risk score was originally applied to 3,142 patients with chronic myeloid leukemia transplanted between 1989 and 1997 in Europe and then was later extended to various diagnoses of hematological disorders undergoing HSCT, including acute myeloid leukemia, acute lymphoblastic leukemia, myelodysplastic syndrome, myeloproliferative disorder, non-Hodgkin lymphoma, multiple myeloma, and aplastic anemia, which had been repeatedly validated in >50000 allogeneic stem cell transplants over ten years [13, 20–23]. The risk score was determined by factors including patient age, disease stage, time interval from diagnosis to transplant, donor types, and donor recipient sex combination [13]. NRM and poor posttransplant OS were all correspondingly higher with increasing EBMT risk score. In some reported articles, they also had investigated the association between the EBMT risk score and OS, leukemia-free survival, and NRM post-HSCT [24–26]. Generally speaking, the EBMT risk score was a good predictor for OS and relapse incidence; however, the scoring system does not include parameters of acute and chronic GVHD [24, 27]. It therefore could not be used to predict the influence of GVHD effect on OS. In our study, we also used EBMT risk score as a way to evaluate pretransplant risk on predicting GRFS. After adjusting for variables including age, hematologic malignancy at diagnosis, gender, conditioning regimen, and posttransplant CMV reactivation, EBMT risk score > 2 was identified as a significant prognostic factor predicting a poor GRFS at either one year or two years (all *p* < 0.001; HR: 1.897 and 1.835 for 1 and 2 years).

It has been claimed that EBMT risk score ≤ 2 could explain more than 60% of the posttransplant 1- or 2-year OS and less than 30% TRM [13, 23]. But the results of OS and TRM could not reflect a real survival without ongoing posttransplant complications. In our study, only less than 40% of patients could survive to 2 years without any complications or death (Figures 1-2) and were significantly and correspondingly lower with increasing EBMT risk score. For EBMT risk score, disease status and time interval between diagnosis and transplantation are associated with disease relapse risk. Risk factors of age, donor types, and sex matching correlate with development of GVHD and the detrimental effect of GVHD in the majority of cases often outweighed the potential benefit of GVL effect. Hence, the aforementioned 3 risk factors are associated with TRM and OS while the RFS is conceivably determined by GVHD-associated GVL effect and intensity of conditioning regimen. To our knowledge, no described scoring system could predict the GRFS currently. The extension of EBMT risk score to GRFS may help us not only integrate the risk stratification of adult allogeneic HSCT but also further assess ideal recovery following HSCT without comorbidity. Our observations warrant a prospective study for further confirmation.

During the period of adult allogeneic HSCT, pretransplant risk factors played an important role for additive impacts on GRFS, but they were not uniform and independent. According to the reported articles, posttransplant CMV reactivation would increase NRM and influence survival in patients receiving allogeneic HSCT [12, 28–30]. In addition, pretransplant comorbidities and quality of life were also important factors for HSCT recipients [31]. Hence, the additional influence of posttransplant CMV reactivation, history of type 2 diabetes mellitus, and smoking history were also evaluated in our study. Although posttransplant CMV reactivation revealed a significantly poor impact on 1- and 2-year GRFS in an univariate analysis, its influence became negligible after adjusting for variables in a multivariate Cox proportional hazards model. On the contrary, GRFS was predominantly poor with increasing EBMT risk scores (scores: 3–7). In conclusion, in the light of presentation of EBMT risk score and specified endpoint analyses of GRFS, EBMT risk scores might present a more objective approach on survival of adult allogeneic HSCT recipients.

Nowadays peripheral blood stem cell has become the predominant stem cell source for HSCT [32, 33] and was associated with inferior GRFS in the report by Holtan et al. [16]. In our patients with a great majority undergoing peripheral blood stem cell transplantation, the 1-year GRFS was a little better than data reported by Holtan et al. [16]. The major events in our patients occurring between 1st and 2nd year were relapse of the malignancies (Figure 3). Within this period, no more new events relating to the toxicities of HSCT, especially acute or chronic GVHD, were noted in our study. These results contribute to a lower NRM. The similar results of GRFS events were also found in the difference of prognostic factors between 1 and 2 years but no increased death rate was noted (Figure 3). These results may explain the reason why we had a better GRFS than the results reported by Holtan et al. However, our retrospective study had some limitations, including less patients receiving bone marrow transplantation and possible underestimation of clinical GVHD in the patients undergoing peripheral blood stem cell transplantation. Besides, more detailed data, such as complete data of HLA-C and DQ, infection type, and cause of death, were lacking and were not included into the analysis of prognostic model.

In conclusion, the GRFS offers an evaluation of real recovery following HSCT and is significantly influenced by some prognostic factors. EBMT risk score can act as a simple and reliable tool to predict GRFS, which provides more information than the traditional measurement of OS or DFS. All these prognostic factors could enhance our ability to optimally judge the risk and the probability of true recovery after allogeneic HSCT in adult patients.

## Figures and Tables

**Table 1:** Clinical characteristics of study patients (*n* = 377).

Patient characteristics	*n*	%
Gender		
Female	168	44.6
Male	209	55.4
Age at HSCT		
≤35	139	36.9
>35	238	63.1
EBMT risk score		
≤2	155	41.1
>2	222	58.9
Indication for HSCT		
AML/MDS	157	41.7
MPD	19	5.0
ALL	74	19.6
Lymphoma	63	16.7
MM	18	4.8
SAA	45	11.9
Others	1	0.3
Donor relation		
Matched sibling	181	48.0
Nonmatched sibling	196	52.0
Conditioning regimen		
TBI-12 Gy based	125	33.2
Fludarabine based	74	19.6
Myeloablative	248	65.8
Type 2 DM history	15	4.0
Smoking history	24	6.4
Posttransplant CMV reactivation		
Within 1 year	188	49.9
Within 2 years	190	50.4
PTLD after HSCT		
Within 1 year	14	3.7
Within 2 years	15	4.0

HSCT: hematopoietic stem cell transplantation; MDS: myelodysplastic syndromes; EBMT: European Group for Blood and Marrow Transplantation; AML: acute myeloid leukemia; MPD: myeloproliferative disorder; ALL: acute lymphoblastic leukemia; MM: multiple myeloma; SAA: severe aplastic anemia; TBI: total body irradiation; CMV: cytomegalovirus; DM: diabetes mellitus; PTLD: posttransplant lymphoproliferative disorder.

**Table 2:** Prognostic factors for 1-year GRFS after adult allogeneic HSCT.

Factors	*n*	Events	%	Univariate analysis			Multivariate analysis		
				HR	95% CI	*p* value	HR	95% CI	*p* value
Age at HSCT									
≤35	139	69	49.6	1.446	1.087–1.923	0.011	1.068	0.783–1.456	0.679
>35	238	151	63.4						
EBMT risk score									
≤2	155	67	43.2	2.197	1.647–2.913	<0.001	1.897	1.385–2.599	<0.001
>2	222	153	68.9						
Gender									
Female	168	84	50.0	1.419	1.081–1.863	0.012	1.310	0.994–1.725	0.055
Male	209	136	65.1						
Disease type									
Nonmalignant	48	16	33.3	2.244	1.348–3.735	0.002	1.763	1.048–2.966	0.033
Malignant	329	204	62.0						
Disease type									
Myeloid	176	111	63.1	0.835	0.641–1.088	0.182
Nonmyeloid	201	109	54.2			
Conditioning									
Nonmyeloablative	129	72	55.8	1.084	0.818–1.436	0.576
Myeloablative	248	148	59.7			
Conditioning									
Others	252	142	56.3	1.212	0.920–1.598	0.172
12 Gy TBI based	125	78	62.4			
Conditioning									
Others	303	168	55.4	1.491	1.092–2.036	0.012	1.134	0.818–1.574	0.450
Fludarabine based	74	52	70.2						
Donor type									
Matched sibling	181	111	61.3	1.005	0.771–1.309	0.971
Others	196	109	55.6			
Type 2 DM									
No	362	210	58.0	1.339	0.710–2.526	0.367
Yes	15	10	66.7			
Smoking history									
No	353	204	57.8	1.348	0.810–2.242	0.250
Yes	24	16	66.7			
Posttransplant CMV reactivation									
No	189	101	53.4	1.335	1.024–1.741	0.033	1.160	0.884–1.522	0.285
Yes	188	119	63.3						
PTLD									
No	363	212	58.4	1.047	0.517–2.120	0.899
Yes	14	8	57.1			

CI: confidence interval; HR: hazard ratio; EBMT: European Group for Blood and Marrow Transplantation; HSCT: hematopoietic stem cell transplantation; PTLD: posttransplant lymphoproliferative disorders; DM: diabetes mellitus; CMV: cytomegalovirus; TBI: total body irradiation.

Factors with statistical significance (*p* < 0.05) upon univariate analysis were included in multivariate analysis.

**Table 3:** Prognostic factors for 2-year GRFS after adult allogeneic HSCT.

Factors	*n*	Events	%	Univariate analysis			Multivariate analysis		
				HR	95% CI	*p* value	HR	95% CI	*p* value
Age at HSCT									
≤35	139	74	53.2	1.452	1.102–1.913	0.008	1.080	0.799–1.459	0.617
>35	238	160	67.2						
EBMT risk score									
≤2	155	73	47.1	2.165	1.640–2.857	<0.001	1.835	1.354–2.486	<0.001
>2	222	161	72.7						
Gender									
Female	168	89	53.0	1.456	1.118–1.896	0.005	1.348	1.032–1.761	0.028
Male	209	145	69.4						
Disease type									
Nonmalignant	48	16	33.3	2.495	1.500–4.149	<0.001	1.979	1.178–3.324	0.010
Malignant	329	218	66.3						
Disease type									
Myeloid	176	117	66.5	0.836	0.647–1.081	0.172
Nonmyeloid	201	117	58.2			
Conditioning									
Nonmyeloablative	129	75	58.1	1.135	0.863–1.494	0.365
Myeloablative	248	159	64.1			
Conditioning									
Others	252	150	59.5	1.246	0.953–1.627	0.107
12 Gy TBI based	125	84	67.2			
Conditioning									
Others	303	179	59.1	1.494	1.104–2.022	0.009	1.115	0.811–1.532	0.503
Fludarabine based	74	55	74.3						
Donor type									
Matched sibling	181	119	65.7	0.976	0.755–1.261	0.851
Others	196	115	58.7			
Type 2 DM									
No	362	224	61.9	1.260	0.699–2.375	0.474
Yes	15	10	66.7			
Smoking history									
No	353	216	61.2	1.415	0.903–2.364	0.122
Yes	24	18	75.0			
Posttransplant CMV reactivation									
No	187	107	57.2	1.349	1.042–1.746	0.023	1.170	0.899–1.523	0.242
Yes	190	127	66.8						
PTLD									
No	362	228	62.9	1.463	0.651–3.292	0.357
Yes	15	6	40.0			

CI: confidence interval; HR: hazard ratio; EBMT: European Group for Blood and Marrow Transplantation; HSCT: hematopoietic stem cell transplantation; PTLD: posttransplant lymphoproliferative disorders; DM: diabetes mellitus; CMV: cytomegalovirus; TBI: total body irradiation.

Factors with statistical significance (*p* < 0.05) upon univariate analysis were included in multivariate analysis.
